# A Novel AVM Calibration Method Using Unaligned Square Calibration Boards

**DOI:** 10.3390/s21072265

**Published:** 2021-03-24

**Authors:** Jung Hyun Lee, Dong-Wook Lee

**Affiliations:** Department of Electronics and Electrical Engineering, Dongguk University, Seoul 04620, Korea; jhlee36@dongguk.edu

**Keywords:** around view monitoring system, automatic camera calibration, vision-based advanced driver assistance systems

## Abstract

An around view monitoring (AVM) system acquires the front, rear, left, and right-side information of a vehicle using four cameras and transforms the four images into one image coordinate system to monitor around the vehicle with one image. Conventional AVM calibration utilizes the maximum likelihood estimation (MLE) to determine the parameters that can transform the captured four images into one AVM image. The MLE requires reference data of the image coordinate system and the world coordinate system to estimate these parameters. In conventional AVM calibration, many aligned calibration boards are placed around the vehicle and are measured to extract the reference sample data. However, accurately placing and measuring the calibration boards around a vehicle is an exhaustive procedure. To remediate this problem, we propose a novel AVM calibration method that requires only four randomly placed calibration boards by estimating the location of each calibration board. First, we define the AVM errors and determine the parameters that minimize the error in estimating the location. We then evaluate the accuracy of the proposed method through experiments using a real-sized vehicle and an electric vehicle for children to show that the proposed method can generate an AVM image similar to the conventional AVM calibration method regardless of a vehicle’s size.

## 1. Introduction

Around view monitoring (AVM) systems eliminate blind spots around the vehicle to prevent car accidents [1]. Because AVM systems create images that show the surrounding view of the vehicle, various vision-based advanced driver assistance systems (ADAS) utilize these AVM-produced images. For example, the parking space detection system detects the parking lines in the AVM images to determine the parking space area [2,3,4], the automated driving system detects the road lanes in the AVM images to track the position of the vehicle [5], and the downward view generation operation transforms an AVM image to generate a downward view image [6]. Therefore, these systems all require well-calibrated AVM images.

**Figure 1:**
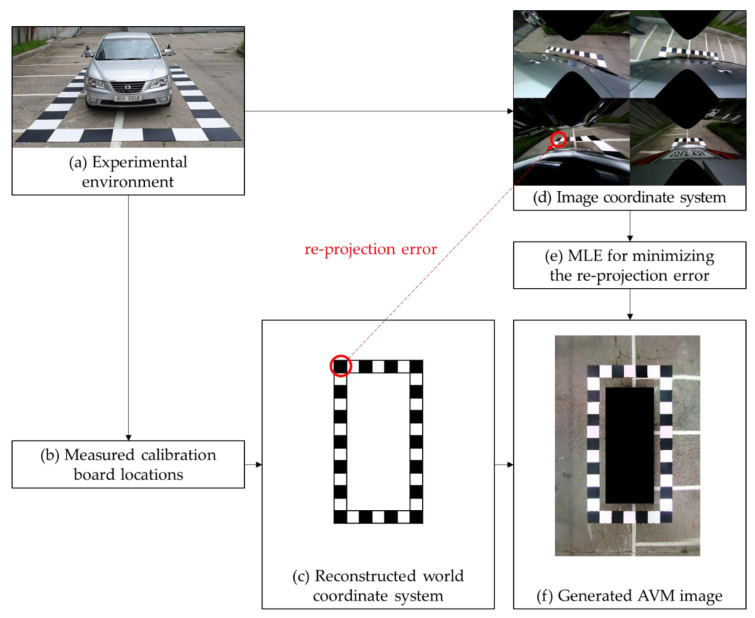
The procedure for conventional around view monitoring (AVM) calibration.

The AVM system transforms four captured images to generate an AVM image, as shown in Figure 1. In AVM calibration, image transformation parameters that are required to generate the AVM images are estimated. These parameters describe the geometrical relationship between the captured image coordinate system and the world coordinate system. In conventional AVM calibration, the maximum likelihood estimation (MLE) is used to estimate this relationship.

The MLE assumes that the location of the calibration board on the surface of the road represents the world coordinate system. Figure 1c shows the reconstructed world coordinate system using the calibration board location. The MLE computes the Euclidean distance, which is the re-projection error between the calibration boards in the reconstructed world coordinate system and the calibration boards in the captured image coordinate system, and minimizes this error to determine the image transformation parameters. Therefore, accurately measuring the calibration board location is a significant and operative procedure of conventional AVM calibration methods to generate well-calibrated AVM images.

**Figure 2:**
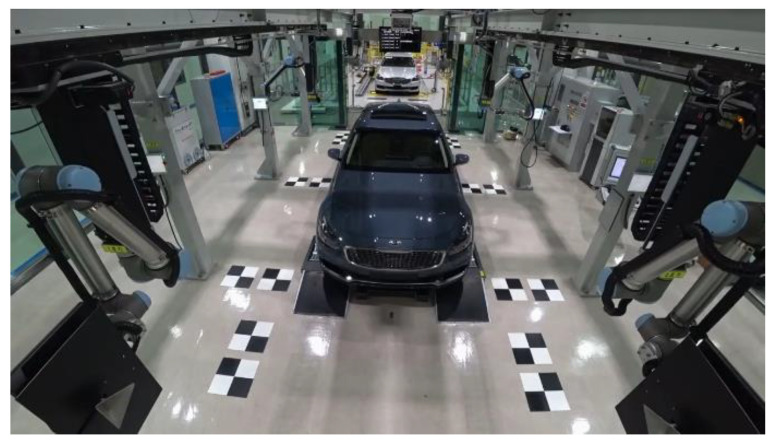
The AVM calibration facility.

Conventional AVM calibration requires the alignment of the calibration boards for measurements, as shown in Figure 1a. Because calibration boards are spread over a large area, accurately measuring calibration boards is an exhaustive procedure. Vehicle manufacturers use AVM calibration facilities to measure the calibration board locations accurately, as shown in Figure 2 [7]. Various AVM calibration studies are also based on well-aligned calibration boards [8,9,10,11,12,13,14,15,16,17,18,19]. The details of these studies are provided in Section 2.1.

Some researchers have utilized alternative devices to facilitate camera calibration [20,21,22,23]. They used odometry or an inertial measurement unit. However, adjacent images utilizing these approach methods cannot be aligned because these methods focus on the calibration of only one camera.

Other approaches detect road lanes or the host vehicle instead of utilizing additional devices [24,25,26,27,28]. These approaches also focus on the calibration of only one camera. Choi et al. [29] calibrated four AVM cameras to align adjacent images using detected road lanes. These calibration methods must repeat the road lane detection process until the integrity of the detected lanes is verified. The methods we have surveyed indicate that camera calibration without the use of calibration boards can face various challenges.

Lee et al. [30] calibrated AVM cameras using only two circle-shaped calibration boards. This method takes multiple photos while the vehicle passes between the two calibration boards to achieve the effect of having more calibration boards placed. However, driving perfectly straight ahead is as exhausting as accurately measuring the calibration board locations. Furthermore, only one calibration board per image with the smallest mean square error is selected from among the multiple images taken while driving. Therefore, this approach is not suitable for the MLE because only one calibration board is used to represent the world coordinate system.

**Figure 3:**
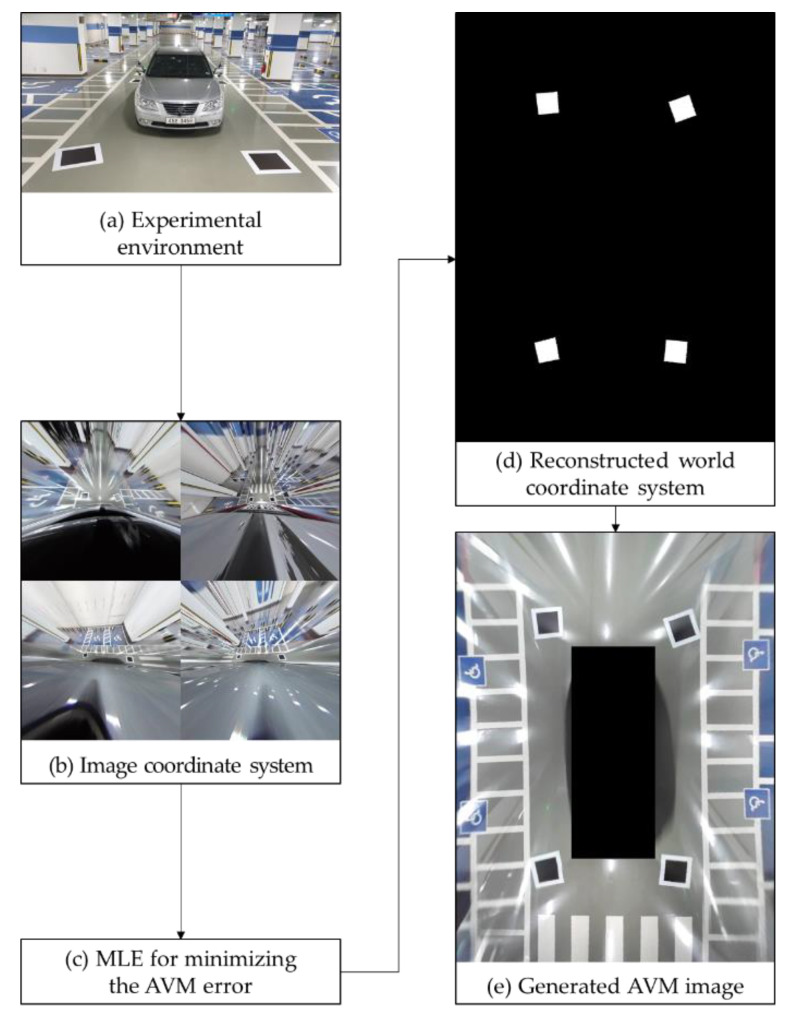
The procedure for the proposed AVM calibration.

**Figure 4:**
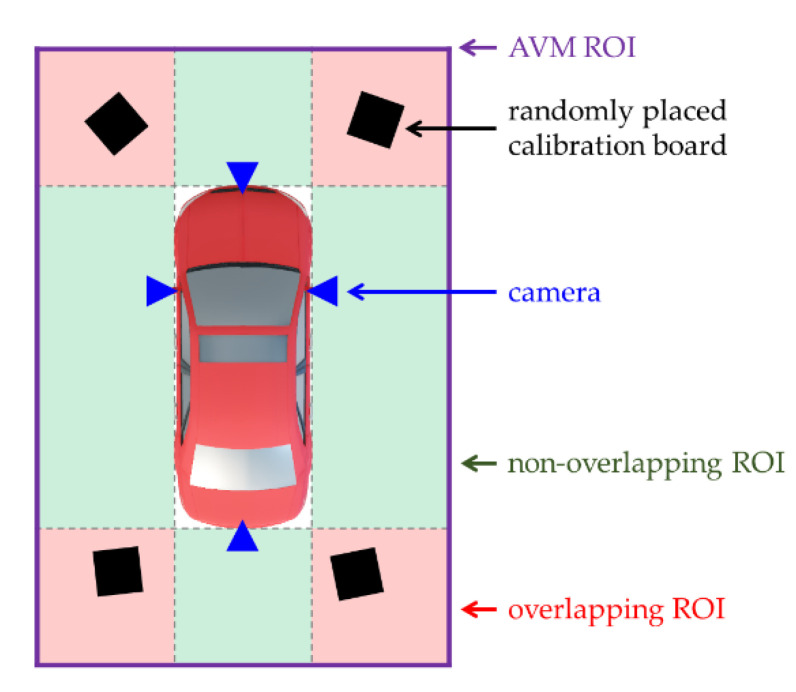
The proposed AVM calibration environment. The region of interest (ROI) of the AVM image (purple rectangle) can be divided into the overlapping ROI (red area) and the nonoverlapping ROI (green area).

We propose an MLE-based AVM calibration method that uses minimal calibration boards, as shown in Figure 3. This method estimates the location of the four calibration boards instead of measuring them. To this end, we divide the AVM image into two areas, as shown in Figure 4. The first area is the overlapping region of interest (ROI) where the fields of view of adjacent cameras overlap. The other area is the nonoverlapping ROI.

At least one calibration board must be placed in each overlapping ROI. If we place additional calibration boards in the nonoverlapping ROI, the accuracy of the MLE will increase. However, the human eye can hardly distinguish between the AVM image results with and without calibration boards placed in the nonoverlapping ROI because the nonoverlapping ROI errors are distributed equally for each pixel and are, therefore, not significant. In contrast, the human eye can easily recognize the overlapping ROI errors because the overlapping ROI is where adjacent images are stitched. Therefore, it is possible to generate an AVM image even if the calibration boards are placed only in the overlapping ROI.

We define two errors to calibrate the AVM cameras using square-shaped calibration boards: a square-shaped error (SSE) and an alignment error (AME). An SSE indicates the difference between the square shape and the quadrilateral shape. A square-shaped calibration board can become a quadrilateral-shaped calibration board in the captured images based on the camera orientation. Therefore, we can estimate the camera orientation by minimizing the SSE. An AME indicates the Euclidean distance between the same calibration boards in the adjacent images. By minimizing the AME, we can estimate the camera position and align the adjacent images. Therefore, we use the sum of the two errors as the loss function of the proposed method.

The proposed AVM calibration offers the following various advantages:

- A measuring procedure is not required.
- Only four calibration boards are used to minimize the placing procedure.
- The proposed method can still generate an AVM image similar to that generated by the conventional method.
- In a small repair shop, the four calibration boards need to be in place only when AVM calibration is being done.

## 2. Related Works

Camera calibration has been extensively researched in a wide range of fields. Therefore, this literature review focuses on two types of AVM calibration-related studies: AVM calibration and vehicle-mounted camera calibration. AVM calibration methods consider the geometric relationship of adjacent AVM cameras. Vehicle-mounted camera calibration methods cannot estimate the adjacent AVM camera relationships, but they can estimate the orientation and position of a mono camera so that these methods can be utilized for AVM calibration.

### 2.1. AVM Calibration

Most of the AVM calibration methods we surveyed use well-aligned calibration boards. Chang et al. [8] proposed a method to determine accurate vertexes of calibration boards when the edges of the calibration boards were blurred and jagged. Zhao et al. [9] reduced the brightness difference among fisheye images and achieved a smooth transition around stitching seam. Two methods [8,9] utilized direct linear transform (DLT) to estimate the image transformation matrix required to generate an AVM image and focused on increasing the accuracy of the AVM calibration.

Gao et al. [10] projected a 2D AVM image generated by the DLT on a 3D ship model. The 3D AVM image helps drivers to be aware of the driving environment and eliminates visual blind spots. Yang et al. [11] proposed a flexible central-around coordinate mapping (CACM) model for vehicle surround view synthesis. The CACM model calculates the geometric relationship between the world coordinate system and the virtual AVM camera coordinate system. These studies focused on mapping models for AVM systems.

Jeon et al. [12] and Lo [13] focused on improving the performance of the embedded system. They also generated an AVM image using the DLT and upload a lookup table including image transformation parameters for generating an AVM image.

No matter how well-aligned calibration boards are used, errors will occur if the coordinates of the calibration boards are not accurately detected in an image. Some researchers proposed a method that can determine the coordinates of calibration boards in an image more accurately. Kim [14] patented a technology for a robot that revises the coordinates of calibration boards in an image. Pyo et al. [15] drew straight lines between calibration boards and detected the vanishing points using the drawn lines. The detected vanishing points help calibration board detection accurately detect the coordinates of calibration boards.

Natroshvili et al. [16] utilized MLE to estimate the orientation and location of cameras. The DLT-based method can only estimate a homography matrix used to transform an image, but the MLE-based method can estimate parameters indicating the orientation and location of cameras. When an AVM image requires revision, adjusting the orientation and location parameters is more intuitive and convenient than adjusting the homography matrix.

Zeng et al. [17] patented an AVM calibration method that paints calibration boards on all grounds, including under the vehicle, to determine the vehicle coordinates accurately. Since the calibration boards under the vehicle are obscured by the vehicle, the coordinates of the vehicle can be estimated.

Ko et al. [18] and Li [19] used a hyperbolic reflector and a spherical image sensor instead of a fisheye lens, respectively. The hyperbolic reflector is a mirror that increases the field of view of a camera by more than 180 degrees. The spherical image sensor can see all 360-degree surroundings by combining two cameras having a field of view of 180 degrees or more.

### 2.2. Vehicle-Mounted Camera Calibration

Camera calibration methods for vehicle-mounted cameras focus on estimating the orientation and location of the camera. The estimated parameters can be used to inverse perspective mapping (IPM). IPM is a method that transforms a captured image into a top view image that removes perspective distortion using the orientation and location of the camera.

Some researchers used additional devices instead of calibration boards. Wang et al. [20] proposed a camera-encoder fusion system to estimate extrinsic parameters. The extracted and tracked natural features provide the Euclidean distance information of the image coordinate system, and the encoder measures the camera travel distance. This method estimates the extrinsic parameters by comparing the Euclidean distance of the natural features with the camera travel distance. Schneider et al. [21] and Chien et al. [22] also measured the camera travel distance using odometry and visual-odometry, respectively. Li et al. [23] used an inertial measurement unit to measure the orientation of the camera.

Other researchers detected road lanes instead of using additional devices or calibration boards. Xu et al. [24] and Prakash et al. [25] detected road lanes and used them for estimating the orientation and location of the front camera. The estimated parameters are used for IPM. A top view image generated by IPM provides the distance between obstacles and the host vehicle. Wang et al. [26] and de Paula et al. [27] also detected road lanes to estimate the orientation and location of a front camera. They estimated the distance between obstacles and the host vehicle without IPM.

Lee et al. [28] proposed a camera calibration method detecting the host vehicle instead of detecting the road lanes. More specifically, this method detects the host vehicle surface to avoid the problems of utilizing detected road lanes, but it can only estimate the orientation of the camera.

## 3. AVM Calibration Using Four Randomly Placed Calibration Boards

The proposed method can generate an AVM image without the location information of the calibration boards. To this end, we estimate the calibration board locations by minimizing the AVM error, which consists of the SSE and AME. In the following sections, we first describe the difference between conventional AVM calibration and the proposed AVM calibration and then define the SSE, AME, and AVM error used to generate an AVM image.

### 3.1. Conventional AVM Calibration

**Figure 5:**
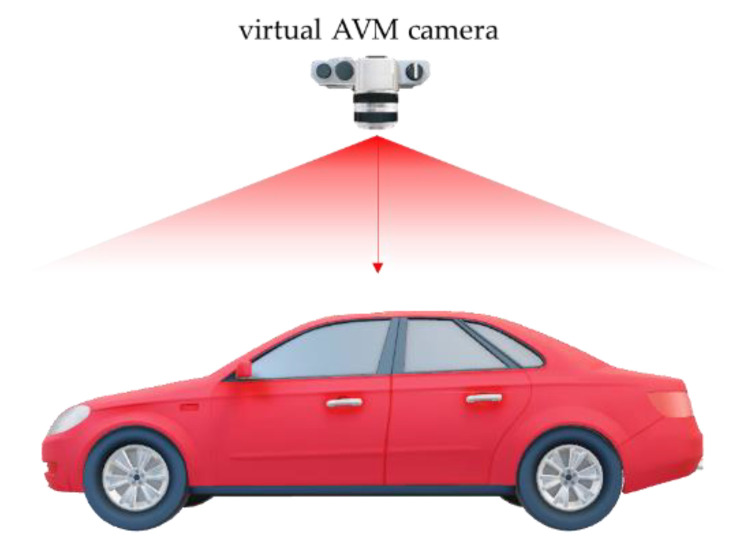
Visualization of the orientation and location of the virtual AVM camera.

The MLE-based conventional AVM calibration estimates the geometrical relationship between the calibration board locations in the world coordinate system and the image coordinate system. Because lens distortion parameters do not change even if the camera orientation and location are changed, we assume that the source images of the AVM calibration are lens distortion-corrected images. The relationship between the world coordinate system and the source image coordinate system can be expressed as follows:

(1) $${\tilde{u}}_{s}=K_{s}\left[R_{s}|T_{s}\right]{\tilde{u}}_{w}$$

where ${\tilde{u}}_{s}$ is the homogeneous source image coordinate system, ${\tilde{u}}_{w}$ is the homogeneous world coordinate system, $R_{s}$ is the rotation matrix describing the camera orientation, $T_{s}$ is the translation matrix describing the camera location, and $K_{s}$ is the intrinsic matrix describing the optical properties of the camera.

(2) $$K_{s}=\left[\begin{array}{cc} f_{s}I_{2\times 2} & p_{s}\\ 0_{2\times 1} & 1 \end{array}\right]$$

where $f_{s}$ is the focal length, $I_{2\times 2}$ is a 2 × 2 identity matrix, and $p_{s}$ is a 2D principal point. We assume that a virtual AVM camera is over the vehicle and looks at the vehicle vertically downward to generate an AVM image, as shown in Figure 5.

**Figure 6:**
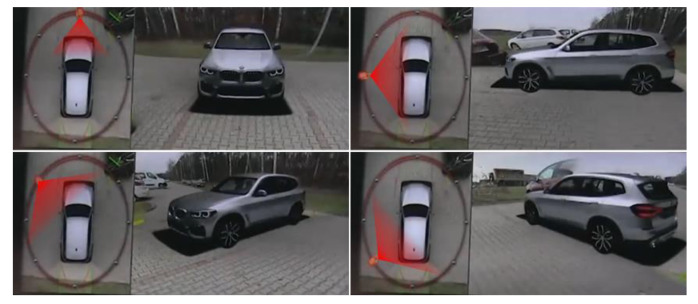
Reconstructed AVM images according to the properties of the virtual AVM camera.

The relationship between the world coordinate system and the AVM image coordinate system can be expressed in the same way as in Equation (1).

(3) $${\tilde{u}}_{v}=K_{v}\left[R_{v}|T_{v}\right]{\tilde{u}}_{w}$$

where ${\tilde{u}}_{v}$ is the homogeneous coordinate system of the virtual AVM image, $R_{v}$ is the rotation matrix describing the virtual AVM camera orientation, $T_{v}$ is the translation matrix describing the virtual AVM camera location, and $K_{v}$ is the intrinsic matrix describing the optical properties of the virtual AVM camera. From Equations (1) and (3), we can express the relationship between the source image coordinate system and the AVM image coordinate system as

(4) $${\tilde{u}}_{v}=\left(K_{v}\left[R_{v}|T_{v}\right]\right){\left(K_{s}\left[R_{s}|T_{s}\right]\right)}^{-1}{\tilde{u}}_{s}=H_{\mathrm{AVM}}{\tilde{u}}_{s}$$

where $H_{\mathrm{AVM}}$ is a 3 × 3 homography matrix describing the relationship between the source image coordinate system and the AVM image coordinate system. The matrix $K_{v}\left[R_{v}|T_{v}\right]$ consists of known parameters because the properties of the virtual AVM camera are determined by the drivers or manufacturers, as shown in Figure 6. Furthermore, because the camera optical properties do not change even if the camera orientation and location are changed, we can assume that the intrinsic matrix $K_{s}$ is known. Therefore, conventional AVM calibration focuses only on estimating the extrinsic matrix $\left[R_{s}|T_{s}\right]$ to compute $H_{\mathrm{AVM}}$. To estimate the extrinsic matrix $\left[R_{s}|T_{s}\right]$, conventional AVM calibration defines a re-projection error $e_{\mathrm{rp}}$ and determines the extrinsic matrix that minimizes the re-projection error.

(5) $$e_{\mathrm{rp}}=\|{\bar{u}}_{v}-H_{\mathrm{AVM}}{\bar{u}}_{s}\|$$

(6) $$\left[{\bar{R}}_{s}|{\bar{T}}_{s}\right]=\underset{\left[R_{s}|T_{s}\right]}{\mathrm{argmin}}\left(e_{\mathrm{rp}}\right)$$

where $e_{\mathrm{rp}}$ is the re-projection error, ${\bar{u}}_{v}$ represents the measured calibration board coordinates for the virtual AVM image coordinate system, ${\bar{u}}_{s}$ represents the measured calibration board coordinates for the source image coordinate system, and $\left[{\bar{R}}_{s}|{\bar{T}}_{s}\right]$ is the estimated extrinsic matrix. Equation (5) is the loss function of the conventional AVM calibration method. Because the calibration board locations are not measured, the measured calibration board coordinates representing the virtual AVM image coordinate system, ${\bar{u}}_{v}$, in Equation (5) is unknown. Therefore, we estimate the calibration board coordinates in the virtual AVM image, ${\bar{u}}_{v}$, to generate an AVM image.

### 3.2. Calibration Board Detection

**Figure 7:**
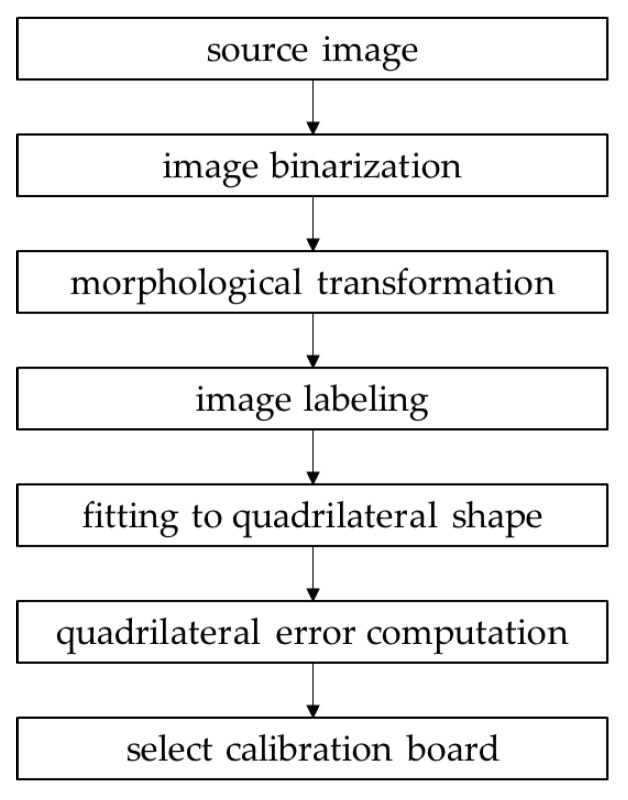
The procedure for calibration board detection.

Calibration board detection occurs in the preprocessing phase of the proposed method. We detect the calibration boards in the source images and utilize them to compute the SSE and AME. Because one calibration board is placed in each overlapping ROI, two calibration boards are photographed in one source image (one source image has two overlapping ROIs). The photographed square-shaped calibration boards become quadrilateral shapes in the source images due to camera tilting. Therefore, we detect two quadrilateral shapes in the source images using simple and commonly used image processing techniques, as shown in Figure 7.

We utilize the adaptive thresholding image binarization method to binarize the source images [31]. This method computes the local threshold values instead of the global threshold value to accurately binarize an image. The morphological transformation can remove noise [32], and the labeling algorithm assigns the pixels to the same group if the values between the neighboring pixels are identical [33]. Next, we detect the edge points of the labeled object and fit the edge points to four straight lines using K-mean clustering [34].

If the labeled object is a quadrilateral, the fitted four straight lines indicate four sides of the quadrilateral. To find the two calibration boards among the labeled objects, we compute the quadrilateral error. The quadrilateral error is the sum of the Euclidean distance between the edge points and the fitted four straight lines. If the labeled object is a quadrilateral, the quadrilateral error is close to zero. Because there are two calibration boards in one source image, we divide the source image into left and right areas and select the labeled object with the least quadrilateral error in each area as the calibration board.

### 3.3. Square-Shaped Error

We can estimate the geometrical relationship between the quadrilateral shape and the square shape because a square-shaped calibration board has a quadrilateral shape in the source image. The square-shaped calibration board can be transformed into a parallelogram shape by an affine transformation matrix, and the parallelogram shape can be transformed into a quadrilateral shape by a perspective transformation matrix.

(7) $$\begin{array}{c} {\tilde{u}}_{\mathrm{parall}}=H_{A}{\tilde{u}}_{\mathrm{square}}=\left[\begin{array}{ccc} a_{11} & a_{12} & 0\\ 0 & 1 & 0\\ 0 & 0 & 1 \end{array}\right]{\tilde{u}}_{\mathrm{square}}\\ {\tilde{u}}_{\mathrm{quad}}=H_{P}H_{A}{\tilde{u}}_{\mathrm{square}}=H_{P}{\tilde{u}}_{\mathrm{parall}}=\left[\begin{array}{ccc} 1 & 0 & 0\\ 0 & 1 & 0\\ p_{31} & p_{32} & 1 \end{array}\right]{\tilde{u}}_{\mathrm{parall}} \end{array}$$

where ${\tilde{u}}_{\mathrm{parall}}$ represents the homogeneous coordinates of the parallelogram-shaped calibration board, ${\tilde{u}}_{\mathrm{square}}$ represents the homogeneous coordinates of the square-shaped calibration board, ${\tilde{u}}_{\mathrm{quad}}$ represents the homogeneous coordinates of the quadrilateral-shaped calibration board, $H_{P}$ is a perspective transformation matrix, and $H_{A}$ is an affine transformation matrix. The parameter $a_{11}$ of the affine transformation matrix $H_{A}$ transforms a square into a rectangle, the parameter $a_{12}$ transforms a rectangle into a parallelogram, the parameter $p_{31}$ of the perspective transformation matrix $H_{P}$ transforms a square into a trapezoid with a parallel pair of opposite sidelines in the horizontal direction, and the parameter $p_{32}$ of the perspective transformation matrix $H_{P}$ transforms a square into a trapezoid with a parallel pair of opposite sidelines in the vertical direction. We can transform the quadrilateral-shaped calibration boards into square-shaped calibration boards with the perspective and affine matrices:

(8) $${\tilde{u}}_{\mathrm{square}}={\left(H_{P}H_{A}\right)}^{-1}{\tilde{u}}_{\mathrm{quad}}$$

To estimate the matrix ${\left(H_{P}H_{A}\right)}^{-1}$ in Equation (8), we define a SSE to indicate the difference between the coordinates ${\tilde{u}}_{\mathrm{quad}}$ and ${\tilde{u}}_{\mathrm{square}}$ using the characteristics of a square shape. The characteristics of a square is that the four angles and the intersection angle of two diagonals are 90 degrees, the length of the four sidelines are equal, and the two diagonals are $\sqrt{2}$ times longer than the sidelines. We define two types of errors based on these characteristics: angle-based SSE (ASSE) and length-based SSE (LSSE). The reason for classifying the SSE into two types is to simultaneously minimize the SSE and AME, details of which are described in Section 3.5.

#### 3.3.1. Angle-Based SSE

An angle-based SSE (ASSE) refers to the difference between an internal angle of a square and the corresponding quadrilateral angle. Let a line vector ${\bar{l}}_{\mathrm{quad},i}$ represent an i-th sideline of a detected quadrilateral-shaped calibration board. By the matrix ${\left(H_{P}H_{A}\right)}^{-1}$ in Equation (8), the detected quadrilateral-shaped calibration board can be transformed into a square-shaped calibration board ${\bar{l}}_{\mathrm{square},i}={\left(H_{P}H_{A}\right)}^{-1}{\bar{l}}_{\mathrm{quad},i}$. The included angle of the square-shaped calibration board can be determined by the dot product of i-th and the j-th line vectors where ${\bar{l}}_{\mathrm{square},i}={\left[\begin{array}{ccc} {\bar{l}}_{1,i} & {\bar{l}}_{2,i} & {\bar{l}}_{3,i} \end{array}\right]}^{T}$.

(9) $$\phi =\cos^{-1}\left(\frac{{\bar{l}}_{1,i}{\bar{l}}_{1,j}+{\bar{l}}_{2,i}{\bar{l}}_{2,j}}{\sqrt{{\left({\bar{l}}_{1,i}\right)}^{2}+{\left({\bar{l}}_{2,i}\right)}^{2}}\cdot \sqrt{{\left({\bar{l}}_{1,j}\right)}^{2}+{\left({\bar{l}}_{2,j}\right)}^{2}}}\right)$$

Therefore, we can define the ASSE as follows:

(10) $$e_{\mathrm{ASSE}}=\left|\frac{\pi }{2}-\phi \right|$$

Equation (10) can be simplified by the cosine function as:

(11) $$\begin{array}{c} e_{\mathrm{ASSE}}=\left|\cos\left(\frac{\pi }{2}\right)-\cos\left(\phi \right)\right|=\left|-\cos\left(\phi \right)\right|\\ =\frac{{\bar{l}}_{1,i}{\bar{l}}_{1,j}+{\bar{l}}_{2,i}{\bar{l}}_{2,j}}{\sqrt{{\left({\bar{l}}_{1,i}\right)}^{2}+{\left({\bar{l}}_{2,i}\right)}^{2}}\cdot \sqrt{{\left({\bar{l}}_{1,j}\right)}^{2}+{\left({\bar{l}}_{2,j}\right)}^{2}}} \end{array}$$

where 0≤ϕ≤π. We then determine the parameters that minimize the ASSE and the calibration boards in the source image can be transformed into square shapes:

(12) $$\left({\bar{H}}_{P},{\bar{H}}_{A}\right)=\underset{H_{P},H_{A}}{\mathrm{argmin}}\left(\overset{2}{\underset{n=1}{\sum }}\overset{5}{\underset{k=1}{\sum }}e_{\mathrm{ASSE}}\left(n,k\right)\right)$$

where $e_{\mathrm{ASSE}}\left(n,k\right)$ is the ASSE of the k-th angle of the n-th calibration board, ${\bar{H}}_{P}$ is the estimated perspective transformation matrix, and ${\bar{H}}_{A}$ is the estimated affine transformation matrix. There are two calibration boards in the source image and five intersection points in the square (four vertices and one center of the square); thus, n is from 1 to 2 and k is from 1 to 5, respectively.

#### 3.3.2. Length-Based SSE

A length-based SSE (LSSE) refers to the sideline length difference between the quadrilateral and square shapes. Let homogeneous coordinates ${\bar{v}}_{\mathrm{quad},i}$ represent the i-th vertex of a detected quadrilateral-shaped calibration board, then the transformed homogeneous coordinates by the matrix variable is ${\bar{v}}_{\mathrm{square},i}={\left(H_{P}H_{A}\right)}^{-1}{\bar{v}}_{\mathrm{quad},i}={\left[\begin{array}{ccc} {\bar{v}}_{1,i} & {\bar{v}}_{2,i} & 1 \end{array}\right]}^{T}$. We can calculate the length of one side using the transformed coordinates ${\bar{v}}_{\mathrm{square},i}$ as:

(13) $${\bar{m}}_{i}=\sqrt{{\left({\bar{v}}_{1,i}-{\bar{v}}_{1,j}\right)}^{2}+{\left({\bar{v}}_{2,i}-{\bar{v}}_{2,j}\right)}^{2}}$$

where ${\bar{m}}_{i}$ is the length of the i-th side of the transformed calibration board. The LSSE can be defined as Equation (14), where the length of one side of the calibration board is m:

(14) $$e_{\mathrm{LSSE}}=\overset{4}{\underset{i=1}{\sum }}\left|m-{\bar{m}}_{i}\right|+\overset{2}{\underset{j=1}{\sum }}\left|\sqrt{2}m-{\bar{d}}_{j}\right|$$

where ${\bar{d}}_{j}$ is the length of the j-th diagonal of the transformed calibration board. We then find the parameters that minimize the LSSE, and the calibration boards in the source image can be transformed into square shapes with:

(15) $$\left({\bar{H}}_{P},{\bar{H}}_{A}\right)=\underset{H_{P},H_{A}}{\mathrm{argmin}}\left(\overset{2}{\underset{n=1}{\sum }}e_{\mathrm{LSSE}}\left(n\right)\right)$$

where $e_{\mathrm{LSSE}}\left(n\right)$ is the ASSE of the n-th calibration board, ${\bar{H}}_{P}$ is the estimated perspective transformation matrix, and ${\bar{H}}_{A}$ is the estimated affine transformation matrix.

### 3.4. Alignment Error

An alignment error (AME) is defined as the Euclidean distance between the same square-shaped calibration boards in adjacent images. Because the quadrilateral-shaped calibration board can be transformed into square-shaped calibration boards by minimizing the SSE, we focus only on estimating the similarity transformation matrix $H_{S}$ consisting of a scale parameter s, an image rotation parameter θ, and image translation parameters $t_{x}$ and $t_{y}$ to align the square-shaped calibration boards in adjacent images.

(16) $$H_{S}=\left[-\begin{array}{ccc} s\cdot \cos\left(\theta \right) & s\cdot \sin\left(\theta \right) & t_{x}\\ s\cdot \sin\left(\theta \right) & s\cdot \cos\left(\theta \right) & t_{y}\\ 0 & 0 & 1 \end{array}\right]$$

Square-shaped calibration boards of a front image and a left image can be aligned using Equation (17).

(17) $$H_{S}^{\mathrm{front}}{\tilde{v}}_{\mathrm{square}}^{\mathrm{front}}=H_{S}^{\mathrm{left}}{\tilde{v}}_{\mathrm{square}}^{\mathrm{left}}$$

where $H_{S}^{\mathrm{front}}$ is the similarity transformation matrix of a front image, and ${\tilde{v}}_{\mathrm{square}}^{\mathrm{front}}$ represents the homogeneous coordinates of the vertex of the square-shaped calibration board of the front image. Therefore, we can define the AME as follows:

(18) $$\begin{array}{c} e_{\mathrm{AME}}=\|H_{S}^{\mathrm{front}}{\bar{v}}_{\mathrm{square}}^{\mathrm{front}}-H_{S}^{\mathrm{left}}{\bar{v}}_{\mathrm{square}}^{\mathrm{left}}\|+H_{S}^{\mathrm{left}}{\bar{v}}_{\mathrm{square}}^{\mathrm{left}}-H_{S}^{\mathrm{rear}}{\bar{v}}_{\mathrm{square}}^{\mathrm{rear}}\|\\ +\|H_{S}^{\mathrm{rear}}{\bar{v}}_{\mathrm{square}}^{\mathrm{rear}}-H_{S}^{\mathrm{right}}{\bar{v}}_{\mathrm{square}}^{\mathrm{right}}\|+\|H_{S}^{\mathrm{right}}{\bar{v}}_{\mathrm{square}}^{\mathrm{right}}-H_{S}^{\mathrm{front}}{\bar{v}}_{\mathrm{square}}^{\mathrm{front}}\| \end{array}$$

where ${\bar{v}}_{\mathrm{square}}$ represents the homogeneous coordinates of the vertex of the transformed calibration boards by the perspective and affine transformation matrices. We can estimate the similarity transformation matrix by minimizing the AME.

(19) $$\left({\bar{H}}_{S}^{\mathrm{front}},{\bar{H}}_{S}^{\mathrm{left}},{\bar{H}}_{S}^{\mathrm{rear}},{\bar{H}}_{S}^{\mathrm{right}}\right)=\underset{\begin{array}{c} H_{S}^{\mathrm{front}}\\ H_{S}^{\mathrm{left}}\\ H_{S}^{\mathrm{rear}}\\ H_{S}^{\mathrm{right}} \end{array}}{\mathrm{argmin}}\left(e_{\mathrm{AME}}\right)$$

where ${\bar{H}}_{S}$ is the estimated similarity transformation matrix.

### 3.5. AVM Error

We can estimate the image transformation parameters for generating the AVM image by minimizing the AVM error, which consists of an SSE and AME. Because there are two types of SSEs, the ASSE and LSSE, the AVM error can be expressed as a combination of the two types: the ASSE–AME and the LSSE–AME.

The problem with the ASSE–AME combination is that the units of the two measurements are not consistent. The ASSE is in radians whereas the AME is in pixels. In contrast, the units for the LSSE and AME are both in pixels. Therefore, we focus on using the LSSE–AME combination. However, the LSSE–AME combination is not without limitations. The LSSE–AME combination suffers from the local minimum problem because the range of the parameters searched by the MLE changes according to the size of the calibration board.

**Figure 8:**
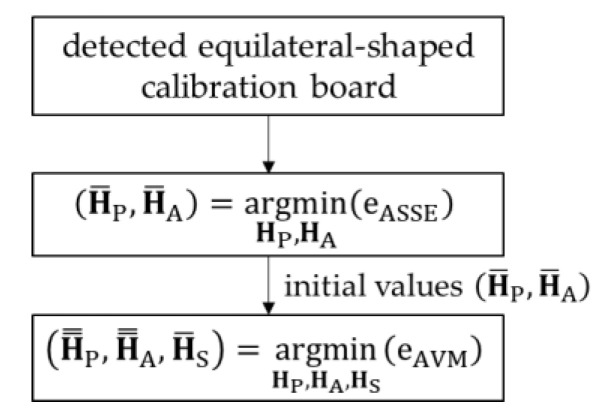
The procedure for the AVM error minimization, where $e_{\mathrm{AVM}}=e_{\mathrm{LSSE}}+e_{\mathrm{AME}}$.

To solve this problem, we find the appropriate initial parameters by minimizing the ASSE. To minimize the ASSE, we utilize the Levenberg–Marquardt algorithm, which is most widely used to solve the maximum likelihood problems of camera calibration. The estimated matrices ${\bar{H}}_{P}$ and ${\bar{H}}_{A}$, by minimizing the ASSE, are used as initial values to minimize the LSSE–AME combination, as shown in Figure 8. Since the matrices ${\bar{H}}_{P}$ and ${\bar{H}}_{A}$ are already optimized, the local minimum problem caused by the size of the calibration board can be solved. The LSSE–AME combination is also minimized by utilizing the Levenberg–Marquardt algorithm.

## 4. Experiments

**Figure 9:**
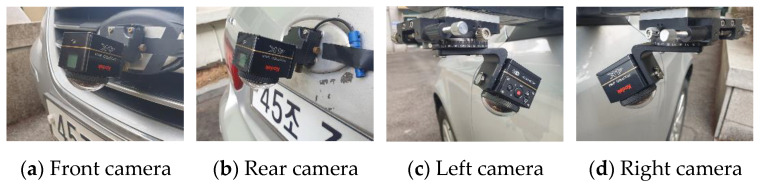
Four installed cameras for the field experiments.

We performed several experiments to evaluate the proposed method. We used Kodak’s PIXPRO SP360 cameras with a 235° field of view and a 2880 px× 2880 px resolution [35]. The cameras were installed on a Hyundai SONATA vehicle, as shown in Figure 9 [36]. The installation heights of the front, rear, left, and right cameras are approximately 57, 84, 92, and 92 cm, respectively. Each camera is tilted approximately 30°. The overall length of the vehicle is 480 cm, the overall width is 183 cm, and the overall height is 147.5 cm. The dimensions of the calibration boards are 50 cm× 50 cm and we set the calibration board dimensions in the AVM image to 100 px× 100 px.

The size of the calibration board must be experimentally determined based on the size of the vehicle and the field of view of the cameras. More specifically, the calibration board size must increase with the increase in the size of the vehicle or the range of the camera field of view. However, the larger the calibration boards, the more inefficient it is to carry and place them. When we used calibration boards with dimensions smaller than 50 cm× 50 cm, sometimes the calibration board detection algorithm failed. When we used calibration boards with dimensions larger than 100 cm× 100 cm, it was difficult to place the calibration boards in the overlapping ROI. Therefore, for the purpose of our experiment, we set the dimensions of the calibration board as 50 cm× 50 cm.

### 4.1. Performance Evaluation Using a Real-Sized Vehicle

**Figure 10:**
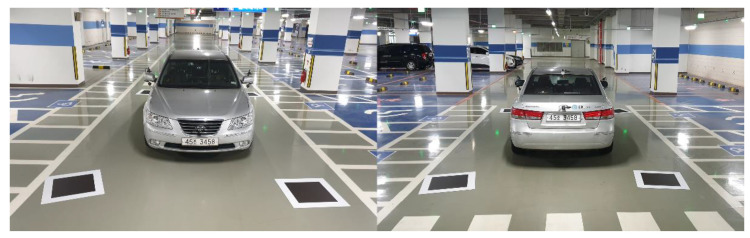
The experimental environment with four randomly placed calibration boards.

**Figure 11:**
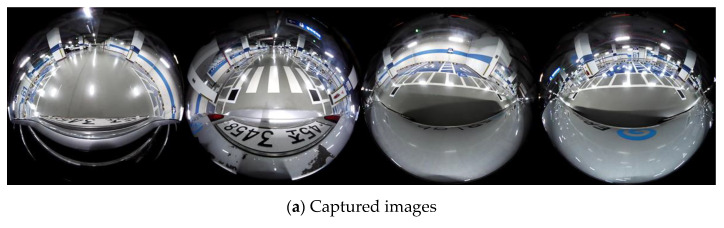

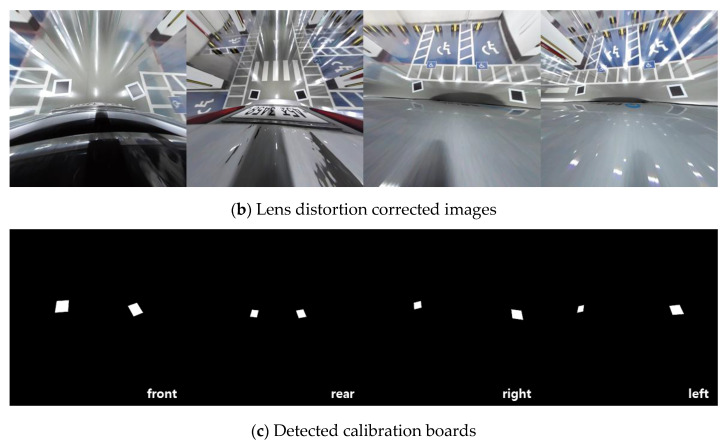
Example images for the performance evaluation of the proposed method.

**Figure 12:**
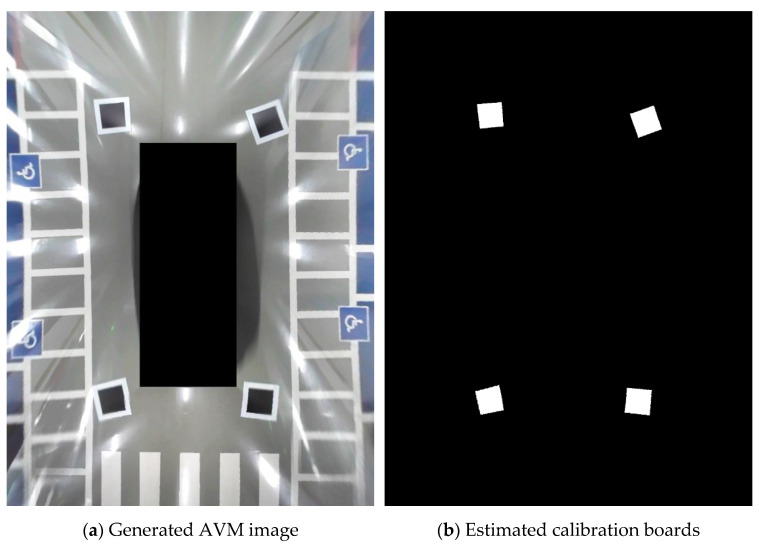
The results of the proposed method.

We placed four calibration boards around the vehicle to evaluate the performance of the proposed method, as shown in Figure 10. Because the camera manufacturer provides the lens distortion parameters and intrinsic parameters, we can easily correct the lens distortion, as shown in Figure 11. In the lens distortion-corrected images, the shape of the calibration boards is quadrilateral. The calibration board detection detects two quadrilaterals per image, as shown in Figure 11c. The proposed method transforms the source images such that the detected quadrilateral calibration boards become squares. Figure 12 shows the generated AVM image using the proposed method. We can observe that all the calibration boards are similar to squares and the adjacent images are well aligned. Furthermore, even though there are no calibration boards in the nonoverlapping ROI, the source image in the nonoverlapping ROI can also be transformed into a well-calibrated AVM image.

**Figure 13:**
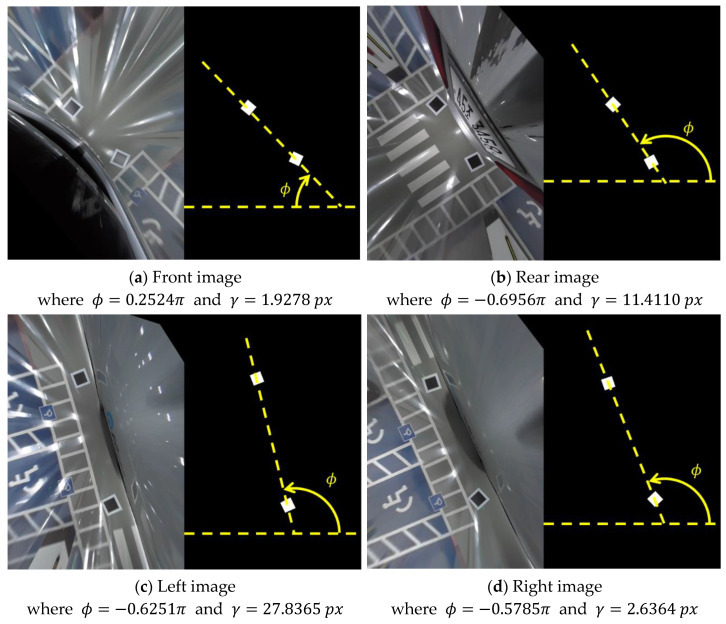
Affine and perspective corrected images, where ϕ is the rotation angle and γ is the scale value by the normalized parameters.

Table 1 shows the estimated image transformation parameters corresponding to the AVM image in Figure 12a. Because the parameters $a_{11}$, $a_{12}$, $p_{31}$, and $p_{32}$ are normalized, the affine and perspective distortion-corrected images are scaled and rotated, as shown in Figure 13. For example, the front image in Figure 13a is rotated 0.2524π clockwise and the average of the side lengths is 1.9278 px when the affine and perspective distortions are corrected. Therefore, the product of s and γ is close to 100 px and the sum of θ and ϕ of the front, left, rear, and right images are close to 0π, −0.5π, −π, and −1.5π, respectively, as shown in Table 2.

For quantitative evaluation, we calculated the AVM errors, as shown in Table 3. Because there are two boards in one image, the LSSE per calibration board is approximately 17.6571/2 = 8.8285 px. The LSSE is the sum of the errors of the four sides and two diagonal lines; thus, the error for each sideline is approximately 8.8285/6 ≈ 1.4714 px. That is, the length of one side of the calibration board is approximately 100 ± 1.4714 px in the generated AVM image. The AME indicates the offset of the adjacent images when two images are stitched. Because one calibration board has four vertexes, the offset of the calibration board is approximately 10.1691/4 ≈ 2.5423 px. These values are significantly small enough to be difficult for the human eye to recognize.

### 4.2. Performance Evaluation Using an Electric Vehicle for Children

**Figure 14:**
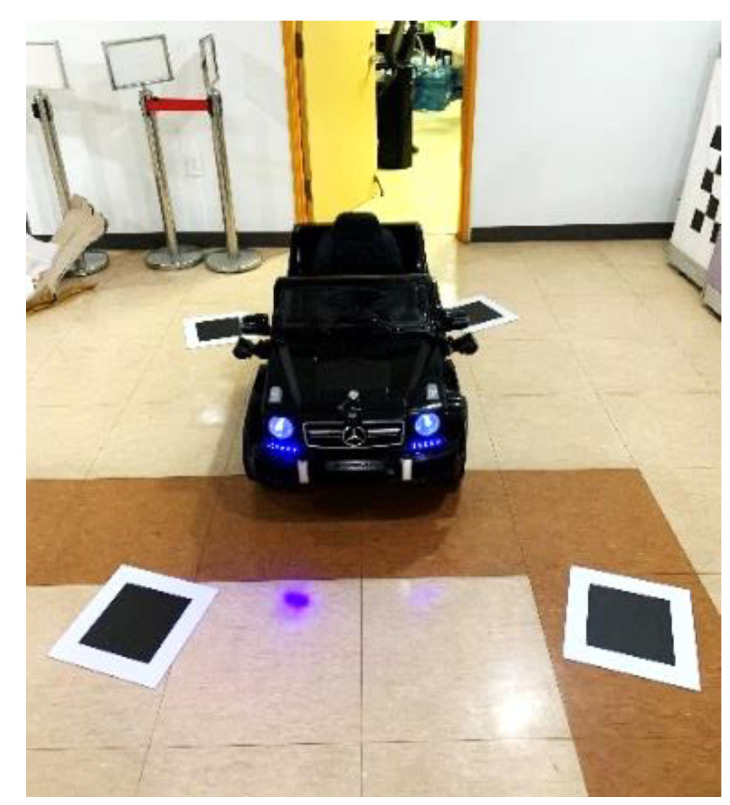
The experimental environment using an electric vehicle for children.

The orientation and location of the camera can change depending on the type and size of a vehicle. Because the proposed method should be able to generate an AVM image regardless of vehicle type, we experimented using an electric vehicle for children to verify this aspect, as shown in Figure 14. The installation height of each camera is approximately 40 cm and each camera is tilted approximately 30°. The overall length of the miniature vehicle is 126 cm, the overall width is 73 cm, and the overall height is 64.5 cm. The dimensions of calibration boards are 20 cm× 20 cm and we set the calibration board dimensions in the AVM image to 100 px× 100 px.

**Figure 15:**
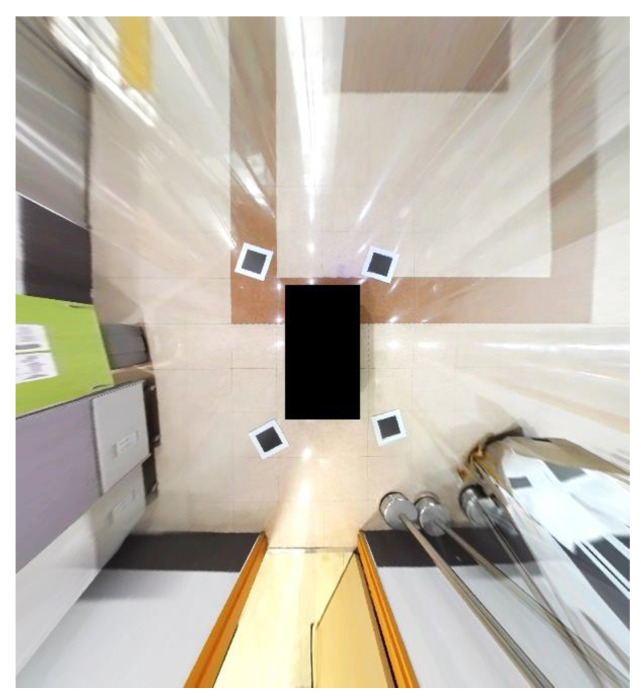
The results of the proposed method using an electric vehicle for children.

Figure 15 shows a generated AVM image using the proposed method for an electric vehicle for children. We can observe that the proposed method can generate a well-calibrated AVM image even though the size of the vehicle is small.

Table 4 shows the calculated AVM errors corresponding to the AVM image in Figure 15. The error for each sideline is approximately 39.5697/12 ≈ 3.2974 px and the offset of the calibration board is approximately 6.6367/4 ≈ 1.6591 px. These resulting values are similar to those of the experimental environment using a real-sized vehicle because the calibration board dimensions in the AVM image are the same in both experiments. From the results of the experiments using real-sized and miniature vehicles, it can be verified that the proposed method can generate an AVM image regardless of the size of the vehicles.

### 4.3. Comparison Experiments with the Conventional Method

**Figure 16:**
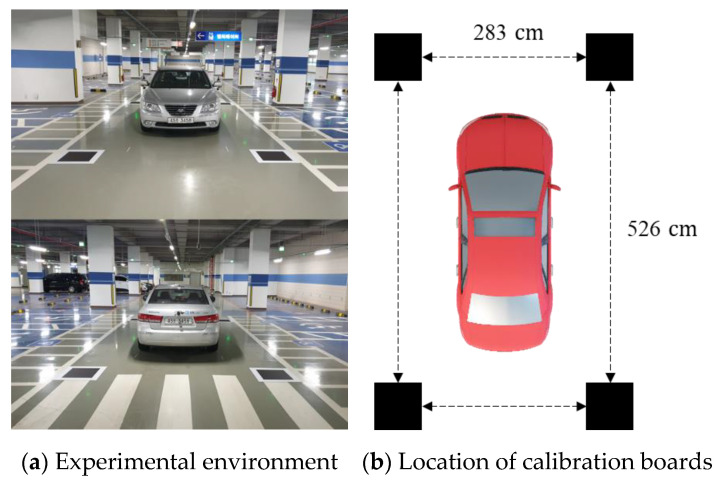
Aligned calibration boards in the conventional method.

The proposed method can generate an AVM image using only four randomly placed calibration boards. In contrast, the conventional methods require calibration boards with known locations. Therefore, to compare the proposed method with the conventional method, we aligned and measured the calibration board locations, as shown in Figure 16, and provided the measured data as input to the conventional method.

**Figure 17:**
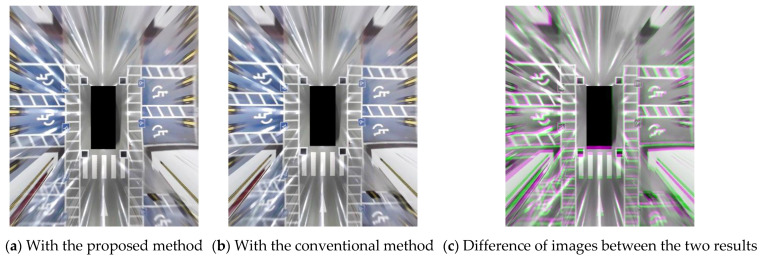
Experimental results of the proposed method and the conventional method. The magenta and green regions show where the grayscale intensities differ.

Figure 17 shows the AVM images generated by the proposed method and the conventional method. We can observe that the results of the two methods are very similar, even though we did not input information regarding calibration board location to the proposed method. To compare the two methods in more detail, we calculated the root mean square error (RMSE), optical flow, and AVM errors for the two AVM images. The RMSE can be expressed as follows:

(20) $$e_{\mathrm{RMSE}}=\sqrt{\frac{1}{mn}\overset{m-1}{\underset{i=0}{\sum }}\overset{n-1}{\underset{j=0}{\sum }}{\left[I_{c}\left(i,j\right)-I_{p}\left(i,j\right)\right]}^{2}}$$

where $I_{c}\left(i,j\right)$ is the grayscale value of the AVM image from the conventional method at the (i,j) point, $I_{p}$ is the grayscale value of the AVM image from the proposed method, m is the width of the AVM images, and n is the height of the AVM images. The calculated RMSE of the two AVM images in Figure 17a,b is 0.0457 when the range of the grayscale is 0–1.

**Figure 18:**
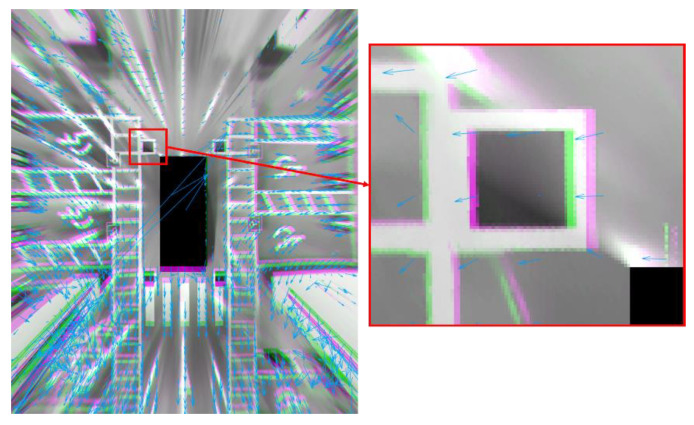
The optical flow between the AVM images of the proposed method and the conventional method, where the blue arrows indicate magnitudes and orientations of the optical flow.

Since the RMSE can depend on the content of the source images, we additionally compute optical flow to measure the displacement. We utilize a method of Farneback [37] to compute optical flow. Figure 18 shows the optical flow between the AVM images of the proposed method and the conventional method. The average of the optical flow is 7.1239 px where the resolution of the AVM image is 1170 px× 1000 px. The RMSE value and the average of the optical flow indicate that the two AVM images are very similar.

Table 5 shows the AVM errors of the proposed method and the conventional method. We can observe that the results of the proposed method are analogous to those of the conventional method. The AVM error in the conventional method is caused by the measurement data error and the calibration board detection error. The AVM error in the proposed method is caused only by the calibration board detection error, not the measurement data error. Therefore, the AVM error in the conventional method is bound to be larger than that of the proposed method.

If we used the AVM calibration facility, the measurement data error would be very small, so the AVM error of the conventional method would have been less or similar to those of the proposed method. However, since we experimented in the same environment without the calibration facility, the AVM error of the conventional method is larger than the proposed method.

These evaluations along with the comparison experiments verify that the proposed method is able to generate an AVM image similar to that of the conventional method without requiring the calibration board location.

## 5. Conclusions

We propose an AVM calibration method using four randomly placed calibration boards and define a novel loss function to utilize the MLE for AVM calibration without the need for information regarding the calibration board locations. The proposed method offers more advantages than the conventional method. The most important advantage is that the proposed method does not require the procedure of measuring the calibration board locations. With this advantage, we can save time and costs that would otherwise be spent on accurately measuring the calibration board locations over a large area. Additionally, as the size of the vehicle increases, the time and cost in using the conventional method also increase, but this is not the case when using the proposed method.

The second advantage of the proposed method is the ability to use the MLE. The most recent AVM calibration method using only two circle-shaped calibration boards cannot utilize the MLE because the MLE requires multiple calibration boards. In contrast, the AVM errors of the proposed method are evenly distributed in all images because we are able to utilize the MLE. The human eye cannot detect the evenly distributed errors.

Flexibility regarding the vehicle size and board size is the third advantage offered by the proposed method. We verify through various experiments that the proposed method can generate AVM images for both real-sized vehicles with large-sized calibration boards and electric vehicles for children with small-sized calibration boards.

Lastly, it is simpler to calibrate AVM systems in the proposed method because there is no need for expert handling facilities for AVM calibration. These advantages were verified through experiments with the vehicle in a parking lot. Based on these advantages, we expect that AVM calibration will be possible in a small repair shop or even in parking lots, resolving the inconvenience of having to visit a large repair shop with AVM facilities for AVM calibration.

## Figures and Tables

**Table 1:** Estimated image transformation parameters.

Parameters	Front	Left	Rear	Right
s	51.8717	3.5862	8.7625	37.9317
θ (rad)	−0.2611π	0.0857π	−0.3056π	−0.8748π
$t_{x}\;\left(px\right)$	−7428.2071	2825.3564	−3613.53	−7673.6563
$t_{y}\;\left(px\right)$	−7496.9527	−2347.6187	−2979.6379	10,336.4311
$a_{11}$	0.0843	0.5115	0.2502	0.1971
$a_{12}$	−0.1002	−0.0410	0.0879	−0.1692
$p_{31}$	−0.0009	−0.0007	−0.0003	−0.001
$p_{32}$	0.0044	−0.0012	−0.0019	−0.0034

**Table 2:** The relationship between the normalized coefficients and estimated parameters.

Parameters	Front	Left	Rear	Right
s	51.8717	3.5862	8.7625	37.9317
γ (px)	1.9278	27.8365	11.4110	2.6364
s×γ (px)	99.9983	99.8273	99.9889	100.0031
θ (rad)	−0.2611π	0.0857π	−0.3056π	−0.8748π
ϕ (rad)	0.2524π	−0.6251π	−0.6956π	−0.5785π
θ+ϕ (rad)	−0.0087π	−0.5394π	−1.0012π	−1.4533π

**Table 3:** AVM errors of the proposed method.

Calibration Board	$e_{\mathrm{AVM}}$	$e_{\mathrm{LSSE}}$	$e_{\mathrm{AME}}$
front-left	25.1992	16.3466	8.8526
left-rear	37.231	25.1189	12.1121
rear-right	24.2864	14.6869	9.5994
right-front	24.5884	14.4762	10.1122
average	27.8262	17.6571	10.1691

**Table 4:** AVM errors of the proposed method using an electric vehicle for children.

Calibration Board	$e_{\mathrm{AVM}}$	$e_{\mathrm{LSSE}}$	$e_{\mathrm{AME}}$
front-left	37.9732	31.5650	6.4082
left-rear	16.0779	8.3447	7.7332
rear-right	34.7484	25.7785	8.9699
right-front	96.0263	92.5907	3.4357
average	46.2065	39.5697	6.6367

**Table 5:** AVM errors in the proposed method and the conventional method.

Calibration Board	$e_{\mathrm{AVM}}$		$e_{\mathrm{LSSE}}$		$e_{\mathrm{AME}}$	
	Proposed	Conventional	Proposed	Conventional	Proposed	Conventional
front-left	20.3643	44.3193	16.5563	26.0181	3.8080	18.3012
left-rear	24.4981	62.4852	20.4678	40.2469	4.0303	22.2383
rear-right	41.5034	57.8872	27.8327	36.4387	13.6708	21.4485
right-front	67.4672	64.4724	64.0037	46.3019	3.4635	18.1706
average	38.4583	57.291	32.2151	37.2514	6.2431	20.0396

## Data Availability

The data presented in this study are available on request from the corresponding author. The data are not publicly available due to ethics.
